# Retrospective Clinical Evaluation of Non-Engaging Abutments Used for Multi-Unit Screw-Retained Fixed Prosthesis

**DOI:** 10.3390/dj13110525

**Published:** 2025-11-10

**Authors:** Paolo De Angelis, Margherita Giorgia Liguori, Edoardo Rella, Davide Piccirillo, Alessandro Donato Tescione, Alberto Staffieri, Paolo Francesco Manicone

**Affiliations:** 1 Division of Prosthodontics, Institute of Clinical Dentistry, Department of Neuroscience, Sensory Organs and Chest, IRCCS Agostino Gemelli Foundation University Hospital, Catholic University of the Sacred Heart, 00168 Rome, Italy; paolo.deangelis@unicatt.it (P.D.A.); davide.piccirillo01@icatt.it (D.P.); alessandro.tescione01@icatt.it (A.D.T.); alberto.staffieri01@icatt.it (A.S.); paolofrancesco.manicone@unicatt.it (P.F.M.); 2 Department of Life, Health and Environmental Sciences, University of L’Aquila, 67100 L’Aquila, Italy; margheritagiorgia.liguori@graduate.univaq.it

**Keywords:** implant dentistry, fixed prosthodontics, screw-retained

## Abstract

**Background/Objectives**: This retrospective study aimed at evaluating the clinical performance of non-engaging abutments in multi-unit implant-supported prostheses and assessing the influence of abutment combinations and clinical variables on biological and technical outcomes. **Methods**: Forty patients with 90 implants and 40 fixed dental prostheses were involved. The study population was divided into three groups: 17 patients (8 males, 9 females) in the first group, 16 patients (7 males, 9 females) in the second, and 7 patients (2 males, 5 females) in the third. All patients received multi-unit implant restorations in a private practice between January 2021 and December 2023, and each prosthesis was in function for one year after delivery, with a mean follow-up of 2.17 ± 0.32 years. Restorations involved non-engaging abutments alone or in combination with engaging abutments or multi-unit abutments. Clinical parameters included implant and prosthesis survival rates, probing pocket depth, bleeding on probing, marginal bone loss, and the prevalence of biological and technical complications. For the comparison between the three groups, the analysis of variance was used to compare group means. When appropriate, Tukey’s post hoc test was applied for multiple comparisons. The significance level was set at *p* < 0.05. **Results**: The implant and prosthesis survival rates were both 100%. Mean marginal bone loss was 0.53 ± 0.33 mm, and mean probing pocket depth was 4.2 ± 0.75 mm. No cases of peri-implantitis were observed. Mucositis prevalence was 11.11% at the implant level. No significant differences were found among different abutment combinations for biological outcomes or complications. The technical complication rate was 5%. **Conclusions**: Non-engaging abutments demonstrated favorable short-term clinical outcomes with minimal biological and technical complications. They may represent a reliable option for the restoration of multi-unit prostheses, although long-term studies are needed.

## 1. Introduction

The implant-abutment connection is a crucial aspect of implant-supported restorations, as it ensures the stability, longevity and biomechanical performance of the whole prosthetic system [1,2,3,4]. Over the years, substantial evidence has demonstrated that implant-abutment connection designs are remarkably precise and durable [1,5,6,7]. The selection of an abutment type must be primarily guided by the clinical scenario, with an important distinction between engaging and non-engaging abutments, each with specific purposes and limitations.

Engaging abutments with a conical connection are designed to lock securely into a conic part of the connection and an anti-rotational interface, such as a hexagon, to provide mechanical stability and resist rotational forces [8]. However, in multi-unit restorations or cases with non-parallel implant placement, engaging base abutments are not a treatment option [8,9].

Non-engaging base abutments are designed with a reduced conical interface and lack the anti-rotational feature [10]. While this characteristic may reduce their ability to resist rotational forces and prevent micro-gaps at the implant-abutment interface, they offer greater flexibility in prosthetic design and alignment corrections. Non-engaging abutments are ideal for challenging clinical scenarios, such as non-parallel implant placements, limited interimplant space and limited tooth-implant space, or angulation discrepancies beyond the tolerance of engaging abutments [11,12,13,14]. They are particularly valuable in multi-unit restorations, facilitating a passive fit and reducing mechanical complications like screw loosening [11,12,13,14,15]. Additionally, their flexibility promotes better stress distribution across the prosthetic framework, potentially improving restoration longevity [16,17,18,19,20,21].

Unfortunately, non-engaging abutments are not lacking limitations. A primary concern is the potential for micro gaps due to mechanical instability at the implant-abutment interface, which can lead to bone remodeling, bacterial colonization [22] and peri-implantitis [23,24,25,26]. Some studies suggest similar microgap sizes for engaging and non-engaging abutments [12], others report higher stress concentrations with non-engaging designs, potentially affecting long-term outcomes [27]. Hybrid solutions, like hemi-engaging fixed dental prostheses (FDPs), combine the stability of engaging abutments with the flexibility of non-engaging ones [20].

To date, although several studies have compared engaging and non-engaging abutments, most have focused on single-unit restorations or in vitro analyses. Limited clinical evidence is available for multi-unit implant restorations in posterior regions. Therefore, this study aimed to evaluate the one-year clinical performance associated with different abutment combinations, focusing on implant and prosthesis survival, marginal bone loss (MBL), probing depth (PPD), biological and technical complications and prosthetic fit. The hypotheses tested were that different non-engaging abutment combinations do not significantly influence survival rates, biological or technical complications, peri-implant bone loss, soft tissue health, or prosthetic fit.

## 2. Materials and Methods

This retrospective cohort study was performed in compliance with the revised Declaration of Helsinki [28]. The protocol was approved by the Ethics Committee of the Catholic University of Sacred Dentistry (Prot. N. 7258; date: 7 January 2025) and followed the Strengthening the Reporting of Observational Studies in Epidemiology (STROBE) guidelines for the conduction and reporting of observational studies [29]. Prior to the participation in the study, all patients read and signed an informed consent document.

The selection involved patients who received a multi-unit implant restoration in private practice between January 2021 and December 2023.

Patients were selected on the basis of the following inclusion criteria: Age > 20 years. Patients who received a multi-unit implant-supported rehabilitation; healthy patients (absence of contraindications for implant surgery and rehabilitation); full-mouth plaque score (FMPS) and full-mouth bleeding score (FMBS) ≤15%; sufficient mesiodistal and interocclusal space; at least one year of follow-up from the functional loading. The full mouth plaque score (FMPS) and full mouth bleeding score (FMBS) were assessed at six sites per tooth (mesio-buccal, mid-buccal, disto-buccal, mesio-lingual, mid-lingual, and disto-lingual) using a periodontal probe. FMPS and FMBS were expressed as percentages, calculated by dividing the number of sites with plaque or bleeding by the total number of sites examined and multiplying by 100.

The exclusion criteria were the following: an American Society of Anesthesiologists (ASA) physical status classification of ≥3. Systemic diseases; untreated periodontal disease; smoking; uncontrolled diabetes; excessive alcohol consumption; incomplete clinical records and radiographs.

All participants who received bone level implants underwent implant placement (type 3) in a prosthetically guided position. From three to six months after the implant placement, the second surgery was performed. When the soft tissue healing was complete, an intraoral scan and a digital workflow were used to create a screw-retained printed provisional restoration. The accuracy of the implant position was verified using an implant verification device. This restoration was used to perform the soft tissue conditioning and a try-on evaluation of function and esthetics. The restoration was finalized delivering a monolithic zirconia (KATANA Zirconia YML, Kuraray Noritake, Singapore) screw retained prosthesis following the manufacturers’ instructions in terms of thickness and surface of the connectors. The passive fit of the final restoration was checked before the delivery using the Sheffield Test. This test, also known as the one-screw test, is an effective clinical method for evaluating the fit of implant-supported frameworks. A framework is considered to fit satisfactorily when tightening a single distal screw does not produce any visible gap between the other abutments [30].

The prostheses were placed on the implants with the screws inserted. Then, starting from the center all the screws were screwed with identical torque. Afterwards, all the screws were removed except for one and an intraoral radiography was taken to assess the passive fit. The abutments used in the study were non-engaging titanium base abutments, engaging titanium base abutments and multi-unit abutments. The abutments were combined according to the clinical situation and the preferences of the operators, always maintaining at least one non-engaging titanium base for each fixed dental prosthesis (FPD). The decisions were made according to several parameters, including the angulation of the implants and relative parallelism, the amount of supra-crestal soft tissues, the occlusal scheme of patients, the available inter-implant space and the height of the prosthesis. At the end of the treatment, all the patients received a nightguard and were enrolled in a six-months maintenance program to perform the hygienic therapy and check the implant (through the peri-implant probing, recording probing pocket depth (PPD) and bleeding on probing (BOP) in 6 sites for each implant) and prosthesis status. Implant placement and prosthetic rehabilitation have been performed by the same two trained appointee operators while follow-up examinations have been performed by a single operator.

The data were collected from the medical records. The following data were gathered as study variables: the demographics of patients (age, gender, date of surgery and of prosthesis delivery), the features of the implants (brand, type of connection and surface, length, diameter), the implant position (anterior, posterior, maxilla, and mandible), type and number of the abutments and their disposition; prosthetic height, number of teeth replaced, number of intermediate elements, periapical radiograph after prosthesis delivery, presence/absence of parafunctions, type of opposing dentition, recall appointments and all type of complications.

The primary outcomes of this study were the implant survival rate and prosthesis survival rate. The secondary outcomes were the prevalence of prosthetic and biologic complications, peri-implant marginal bone loss, probing pocket depth and bleeding on probing. The implant survival and prosthesis survival were defined as the implant or prosthesis remaining in situ. The technical complications, as well as the biological ones, were retrieved from the clinical chart of the patients.

Technical complications were divided into minor and major complications. The minor ones were considered those easily repairable with a chairside approach. The major complications were identified as those requiring additional laboratory procedures and/or components.

The biological complications were divided into mucositis and peri-implantitis analyzing the probing pocket depth, bleeding on probing, suppuration and the peri-implant marginal bone loss. The peri-implant marginal bone loss was radiographically measured using periapical radiographs and the parallel technique. The peri-implant marginal bone loss was measured with an imaging software by the same operator (P.D.A.) after an intra-examiner calibration and it was defined as the distance in millimeters between the shoulder of the implant and the first bone-to-implant contact recording the highest value, either mesial or distal.

Categorical variables were presented as absolute and relative frequencies, while numerical variables were expressed as mean ± standard deviation and/or median with minimum and maximum values. The Shapiro–Wilk test and visual inspection of distribution plots were used to assess normality. For the comparison between the three groups, the analysis of variance (ANOVA) was used to compare group means. Specifically, a one-way ANOVA was performed to assess the effect of the surgical approach to several dependent variables. When appropriate, Tukey’s post hoc test was applied for multiple comparisons. The significance level was set at *p* < 0.05. The data were analyzed using STATA 18 (StataCorp. 2023. Stata Statistical Software: Release 18). Descriptive data related to patients and implants were summarized as counts and percentages. The results are presented as odds ratios with 95% confidence intervals (CIs). A two-sided *p* < 0.05 was considered statistically significant.

## 3. Results

A total of 40 patients were included in the study, comprising 23 females (57.5%) and 17 males (42.5%), with a mean age of 44.97 ± 12.08 years (range: 24–72) (Table 1). All patients received multi-unit implant restorations in a private practice between January 2021 and December 2023, and each prosthesis was in function for at least one year following delivery, with a mean follow-up of 2.17 ± 0.32 years.

A total of 90 implants and 40 fixed prostheses were employed: 17 (42.5%) were located in the posterior mandible, 2 (5%) in the anterior mandible and 16 (40%) were placed in the posterior maxilla, while 5 (12.5%) in the anterior. The length of implants ranged from 8 to 12 mm and the diameter of implants ranged from 3.75 to 5 mm.

**Figure 1:**
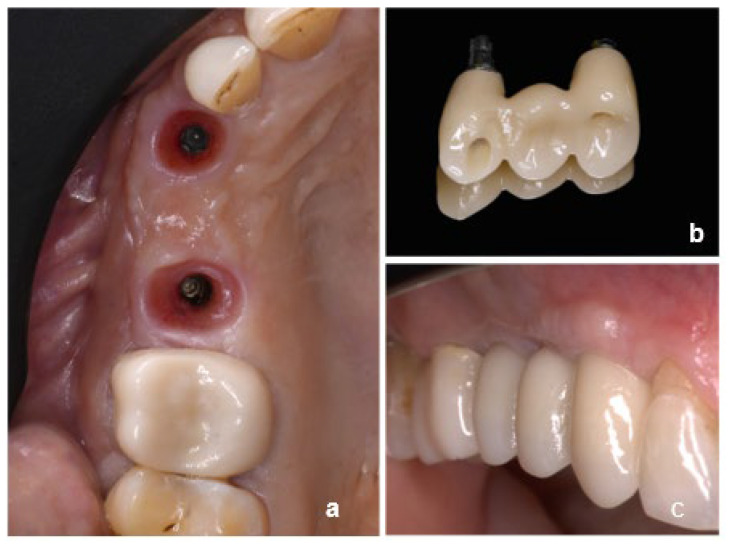
Clinical case showing the combination of non-engaging and engaging abutments: (**a**) occlusal view of peri-implant soft tissues, (**b**) implant-supported prosthesis, and (**c**) intraoral view of the final restoration.

**Figure 2:**
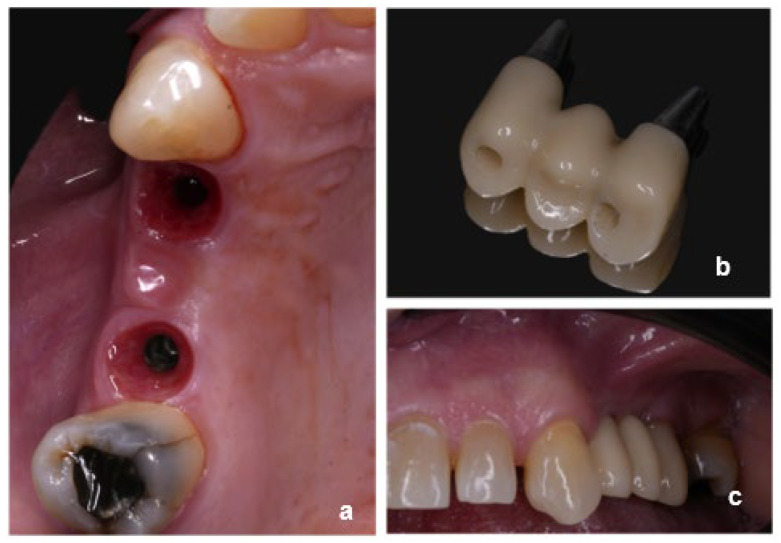
Clinical case showing non-engaging abutments: (**a**) occlusal view of peri-implant soft tissues, (**b**) implant-supported prosthesis, and (**c**) intraoral view of the final restoration.

**Figure 3:**
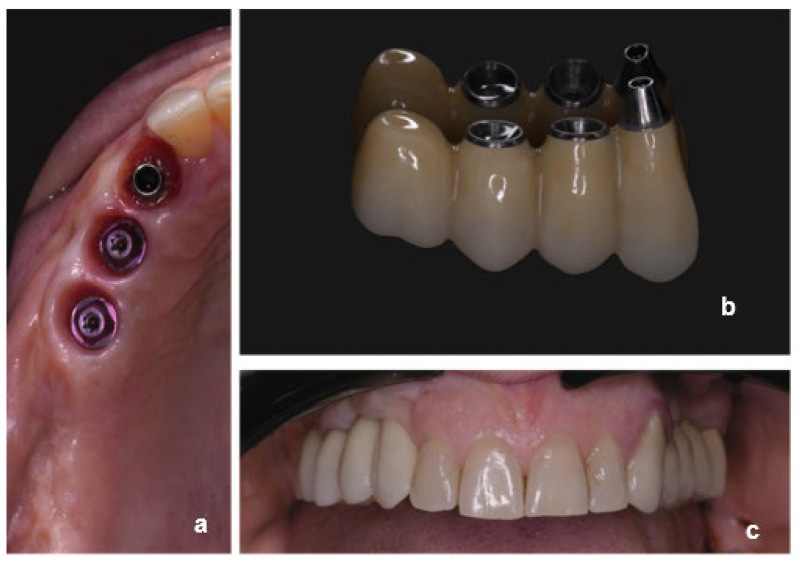
Clinical case showing the combination of non-engaging and multi-unit abutments: (**a**) occlusal view of peri-implant soft tissues, (**b**) implant-supported prosthesis, and (**c**) intraoral view of the final restoration.

Among all, 16 cases were finalized using non-engaging abutments associated with engaging abutments (Figure 1) (one case using a non-engaging abutment with an angulated screw channel), 17 cases were delivered using only non-engaging abutments (Figure 2) and seven were delivered combining non-engaging abutments and multi-unit abutments (Figure 3). The number of intermediate elements of the FDPs ranged from 1 to 3.

Two implants failed before the prosthetic loading and were removed during the second surgery, due to mobility resulting in an early failure. No late implant failures were recorded.

**Figure 4:**
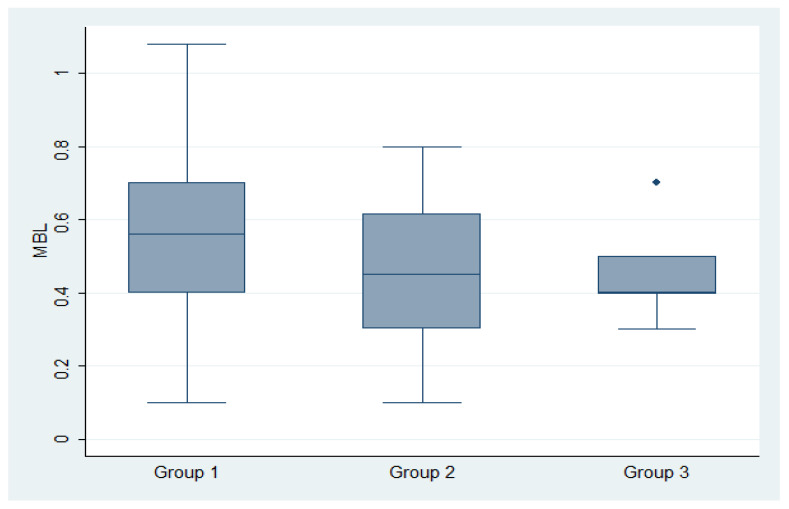
Box plots of the marginal bone loss (MBL) in group 1 (non-eng/eng), group 2 (non-eng) and group 3 (non-eng/multi-unit).

**Figure 5:**
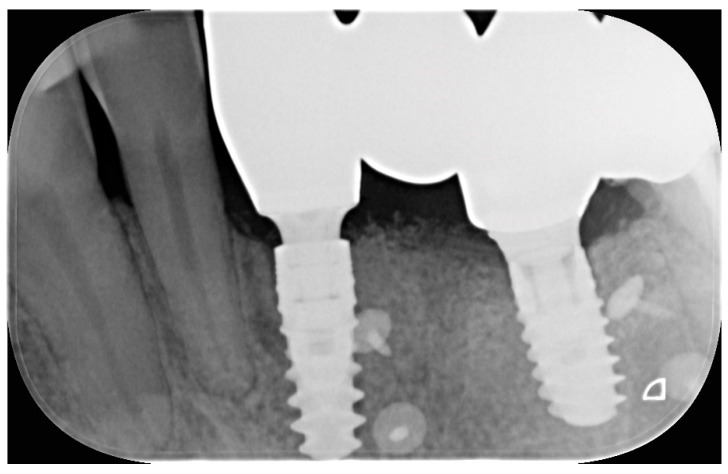
A peri-apical radiograph showing one of the included rehabilitations.

The mean MBL was 0.53 ± 0.33 (Figure 4 and Figure 5) and the mean PPD was 4.2 ± 0.76 one year after the loading with non-statistically significant differences from the prosthesis delivery (*p* > 0.05). The implant survival rate for the first year of function was 100% at implant level. Ten implants were affected by mucositis.

At implant level, peri-implant mucositis prevalence was 11.11%. At patient level, peri-implant mucositis was 6.66% (Table 2).

No cases of peri-implantitis were recorded a year from the loading. No statistically significant differences were recorded among the different clinical combinations of non-engaging and other abutments for the biological complications prevalence (*p* = 0.97), MBL (*p* = 0.23), PPD (*p* = 0.27).

The prosthesis survival rate was 100%. Two events, classified as minor complications, were recorded during the follow-up and no major complications occurred. One of the complications was a screw loosening occurred nine months after the follow-up in the group non-engaging/non-engaging in a case with augmented prosthetic height and parafunction. The other complication was represented by the non-engaging base decementation after two months in the group engaging/non-engaging. The overall rate of technical complications was 5%. During the prosthesis delivery, no clinically and radiographically detectable differences were observed in terms of fitting discrepancy, among the different abutment combinations.

## 4. Discussion

This retrospective clinical study evaluated the efficacy and performance of non-engaging abutments in multi-unit implant-supported prostheses. These abutments, designed to promote a more passive fit, were assessed with respect to mechanical complications, prosthetic fit and overall clinical outcomes. The results suggest that non-engaging abutments are not associated with clinical and radiographic complication at the one year follow up.

One of the key findings of this study is the reduced incidence of mechanical complications, such as screw loosening, in the non-engaging group. These events are frequently associated with stress at the implant–abutment interface, caused by misfit or micromovements, which are more commonly observed with engaging abutments [31,32]. This is aligned with the previous literature, highlighting the importance of passive fit for the long-term success of implant-supported restorations [32,33]. The improved seating precision and reduced strain on peri-implant tissues further support these findings [34].

The results indicate that non-engaging abutments are suitable for multi-unit prostheses, where the need for passive fit and uniform stress distribution is paramount. Engaging abutments, known to offer mechanical retention and prevent rotational movement in single-unit restorations, may introduce unnecessary stress when used across multiple implants [12,19]. This additional stress can contribute to long-term complications, such as peri-implant bone loss, which has been recognized as a risk factor for implant failure [35]. The current study corroborates the above by showing that the use of non-engaging abutments in multi-unit prostheses can mitigate these risks, highlighting the need to carefully select the appropriate abutment design, based on the clinical scenario [36].

Several studies support the clinical rationale for the use of non-engaging abutments in multi-unit cases. Schoenbaum et al. suggested that hemi-engaging designs may improve handling but that fully non-engaging abutments reduce mechanical complications in longer-span prostheses [20].

Alzoubi et al. reported no significant differences in microgap size (≤10 µm versus >10 µm) between engaging and non-engaging designs, indicating that the connection geometry may not be the main factor affecting marginal adaptation [12]. Additionally, both Sakar et al. and Byun et al. demonstrated the improved stress distribution with non-engaging abutments, supporting their use in reducing mechanical overload [19,33]. These data reinforce the relevance of prosthetic fit and stress control in long-term outcomes.

As already underlined, the reduction in stress at the implant–abutment interface is not only relevant for the prevention of mechanical complications but may also play a role in preserving marginal bone levels. Excessive or uneven stress distribution has been associated with peri-implant bone loss, especially in the presence of prosthetic misfit or inadequate passivity [37]. A multi-unit comparative test evaluated non-engaging abutments under ISO 16256 (Clinical Laboratory Testing and In Vitro Diagnostic Test Systems—Broth Micro-Dilution Reference Method for Testing the In Vitro Activity of Antimicrobial Agents Against Yeast Fungi Involved in Infectious Diseases, 2021) [38]. and showed higher compressive and fatigue strength than the comparator system, while SEM cross-sections confirmed implant–abutment gaps ≤10 µm after 5 million cycles. Although clinical MBL was not measured, this combination of superior fatigue resistance and preserved fit supports the expectation of limited early crestal remodeling with non-engaging abutments in splinted prostheses [39].

In addition to mechanical factors, the frequency of abutment disconnections may influence peri-implant tissue stability. With non-engaging abutments, prosthesis removal represents a significant disadvantage because it inevitably requires abutment disconnection, whereas multi-unit abutments are designed to remain in place, avoiding repeated manipulation of the implant–abutment interface. Maintaining a stable abutment, following the “one abutment–one time” approach, has been shown to positively affect peri-implant bone remodeling during the first year of loading, whereas repeated disconnections may disrupt the mucosal seal, leading to apical displacement of connective tissue and increased marginal bone loss [40,41].

A more recent randomized clinical trial compared the one-time placement of the definitive abutment at functional loading to a protocol in which the healing abutment was disconnected and reconnected multiple times during the prosthetic phase. Although both protocols showed statistically significant bone remodeling (marginal bone loss of approximately 0.5 mm at 6 and 12 months), no significant differences were observed in clinical parameters, including probing depth, full-mouth plaque and bleeding scores, or patient-reported outcomes [41]. These findings suggest that, while minor statistical differences may exist, they do not translate into clinically meaningful differences, indicating that both approaches provide comparable outcomes in terms of peri-implant tissue stability and prosthetic function. Finally, clinical factors such as implant position and depth, as well as the dimensions or morphology of the multi-unit abutments, may influence the selection of abutments for screw-retained restorations, often favoring non-engaging base abutments in specific scenarios. Similarly, platform-switching connections can have a role on the amount of marginal bone loss, which is strictly related to the connection design and can therefore change between different implants. Our results show a slight MBL which is similar to what was found in other articles employing similar abutment designs; this shows that t-bases (both engaging and non-engaging) connection to implants is strong enough to reduce the MBL directly linked to micromovements of the crown-abutment complex [42].

In terms of biological complications, no cases of peri-implantitis were observed among the included restorations. Mucositis was recorded, but the prevalence of this condition did not significantly differ among the various clinical combinations of non-engaging and other abutments, as reported by previous studies [43]. The mean probing depth was slightly higher than typically reported, likely due to multi-unit prosthesis design, probing at six sites per implant, and individual tissue variability. Nevertheless, despite the presence of mucositis, all implants remained stable without progression to peri-implantitis.

These findings support the assumption that abutment geometry, when proper hygiene protocols and prosthetic contours are respected, may not be the primary determinant of biological outcomes [44,45].

In the present study, implants restored using non-engaging abutments demonstrated a 100% survival rate, with no prosthetic failures reported. From a clinical perspective, these abutments may also contribute to simplified treatment protocols by reducing the number of components and adjustments needed. In addition to mechanical advantages, non-engaging abutments may offer practical benefits in terms of restorative space. Their simplified design results in a more compact vertical and horizontal profile compared to multi-unit abutments. The design simplification can be advantageous in cases with limited vertical and horizontal restorative space, offering improved flexibility in prosthetic contours and emergence profile design. Although clinical experience suggests that non-engaging abutments may require less restorative space than multi-unit abutments, further studies are needed to confirm this observation. Furthermore another critical issue is related to the decontamination and sterilization protocol of the restoration and the bases after the extra-oral cementation and the intra-oral try in with the risk of surface modification and contamination during the manipulation phases.

The present study provides new clinical data on multi-unit implant restorations by directly comparing engaging and non-engaging abutments in posterior regions. Unlike most previous reports, which have focused on single crowns or laboratory analyses, our findings offer real-world clinical evidence on the short-term functional and mechanical behavior of these abutment types. However, certain limitations should be acknowledged. The relatively short follow-up period may not be sufficient to detect late-emerging biological or technical complications. Moreover, the retrospective design may introduce variability in clinical factors, including implant number, distribution, and abutment combinations, which could influence the outcomes and limit the generalizability of the results. Additionally, the small sample size in some subgroups may have reduced the statistical power to detect differences in certain outcomes. This limitation is inherent to the retrospective design and should be considered when interpreting the results. Future prospective studies with longer observation periods, larger and more balanced sample sizes, and standardized treatment protocols are warranted to confirm these findings and to further evaluate the long-term performance and predictability of non-engaging abutments across different clinical scenarios.

## 5. Conclusions

Within the limitations of this retrospective study, non-engaging abutments demonstrated favorable clinical performance in multi-unit implant-supported prostheses after one year of function. The low incidence of technical complications and the absence of detectable prosthetic misfit suggest that non-engaging abutments, alone or in combination, may be a reliable option for the restoration of multi-unit prostheses. However, given the limited available evidence and the short follow-up, these findings should be interpreted with caution, and further long-term studies are needed to confirm these results over extended periods.

## Figures and Tables

**Table 1:** Sample characteristics.

	*n* = 40
Age (Mean ± sd)	44.97 ± 12.08
Gender (%)	17 M (42.5%) 23 F (57.5%)
PPD (Mean ± sd)	4.20 ± 0.76
MBL (Mean ± sd)	0.53 ± 0.33

PPD = probing pocket depth; MBL = marginal bone loss.

**Table 2:** Sample characteristics divided by group.

	Group 1 (*n* = 17)	Group 2 (*n* = 16)	Group 3 (*n* = 7)
Age (Mean ± sd)	46.07 ± 13.15	47.03 ± 11.43	37.60 ± 9.09
Gender (%)	8 M (47%) 9 F (53%)	7 M (44%) 9 F (56%)	2 M (29%) 5 F (71%)
PPD (Mean ± sd)	4.06 ± 0.83	4.44 ± 0.73	4.02 ± 0.58
MBL (Mean ± sd)	0.64 ± 0.44	0.46 ±0.21	0.44 ± 0.13

Group 1: non-eng/eng; Group 2: non-eng; Group 3: non-eng/multi-unit. PPD = probing pocket depth; MBL = marginal bone loss.

## Data Availability

The raw data supporting the conclusions of this article will be made available by the authors on request.
